# Supplementary material for: Tead1a Initiates Transcriptional Priming Through the TEAD1a/YAP‐Notch1‐Spi1/Cebpα Axis to Promote Neutrophil Fate
Source: Adv Sci (Weinh). 2025 Aug 28;12(41):e05441. doi: 10.1002/advs.202505441 (PMC12591127; doi:10.1002/advs.202505441)
Supplement: Supplementary file 4 — Supplementary Dataset [file ADVS-12-e05441-s004.zip › 3-7.zip!/3-7/02-7.cluster/cluster18/Enrich/ego_MF.pdf]

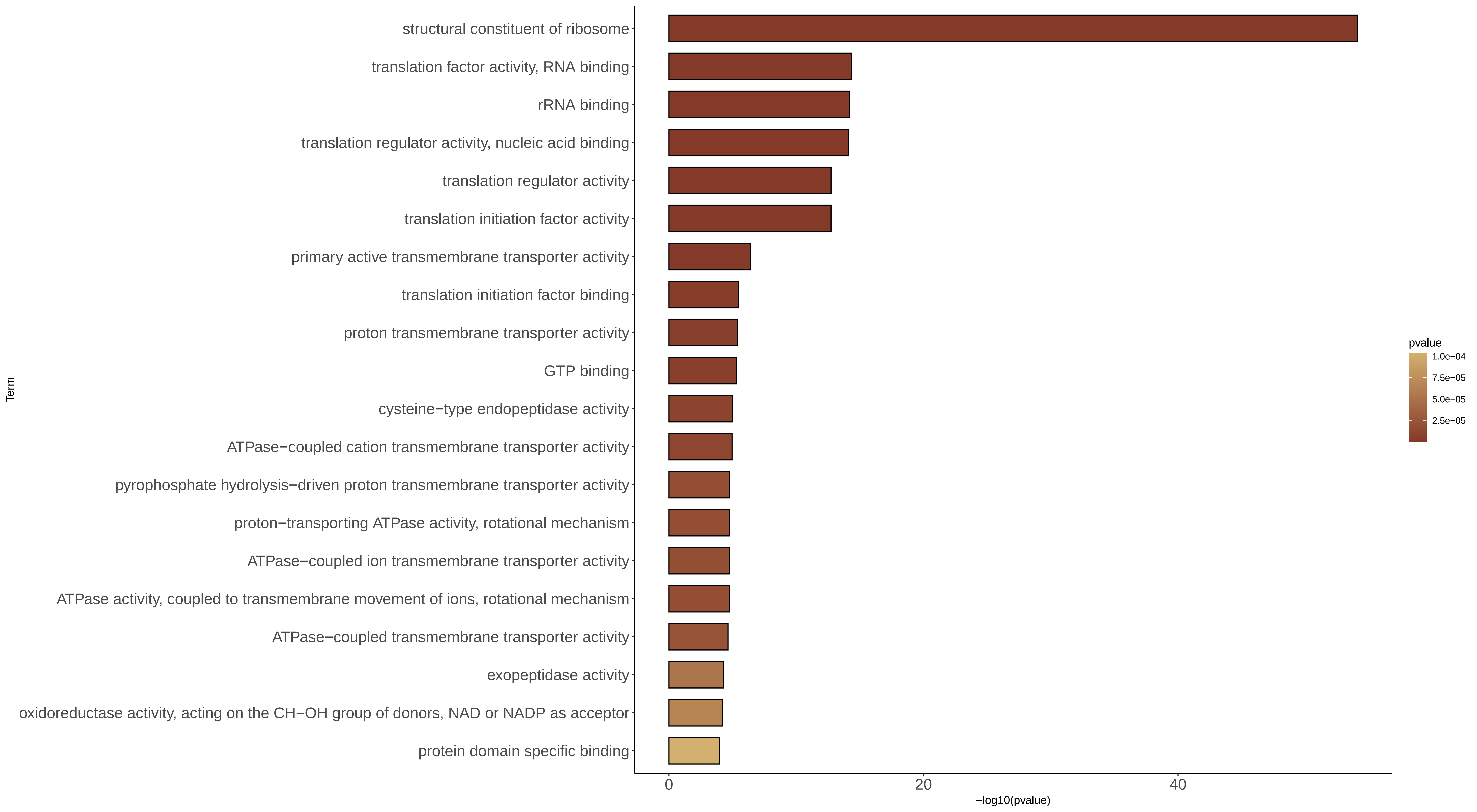
structural constituent of ribosome
translation factor activity, RNA binding
rRNA binding
translation regulator activity, nucleic acid binding
translation regulator activity
translation initiation factor activity
primary active transmembrane transporter activity
translation initiation factor binding
proton transmembrane transporter activity
GTP binding
cysteine−type endopeptidase activity
ATPase−coupled cation transmembrane transporter activity
pyrophosphate hydrolysis−driven proton transmembrane transporter activity
proton−transporting ATPase activity, rotational mechanism
ATPase−coupled ion transmembrane transporter activity
ATPase activity, coupled to transmembrane movement of ions, rotational mechanism
ATPase−coupled transmembrane transporter activity
exopeptidase activity
oxidoreductase activity, acting on the CH−OH group of donors, NAD or NADP as acceptor
protein domain specific binding
Term
0
20
40
−log10(pvalue)
pvalue
1.0e−04
7.5e−05
5.0e−05
2.5e−05
